# Impact of Retinopathy and Systemic Vascular Comorbidities on All-Cause Mortality

**DOI:** 10.3389/fendo.2021.750017

**Published:** 2021-11-18

**Authors:** Zhuoting Zhu, Xianwen Shang, Wei Wang, Jason Ha, Yifan Chen, Jingyi He, Xiaohong Yang, Mingguang He

**Affiliations:** ^1^ Department of Ophthalmology, Guangdong Eye Institute, Guangdong Provincial People’s Hospital, Guangdong Academy of Medical Sciences, Guangzhou, China; ^2^ Ophthalmology, Department of Surgery, Centre for Eye Research Australia, University of Melbourne, Melbourne, VIC, Australia; ^3^ State Key Laboratory of Ophthalmology, Zhongshan Ophthalmic Center, Sun Yat-sen University, Guangzhou, China; ^4^ Medical Sciences Division, University of Oxford, Oxford, United Kingdom; ^5^ Zhongshan School of Medicine, Sun Yat-sen Universtiy, Guangzhou, China

**Keywords:** retinopathy, systemic vascular comorbidities, diabetes, hypertension, kidney disease, cardiovascular disease, mortality

## Abstract

**Purpose:**

To assess the impact of retinopathy and systemic vascular comorbidities on the all-cause mortality in a representative U.S. sample.

**Methods:**

A total of 5703 participants (≥40 years old) from the 2005-2008 National Health and Nutrition Examination Survey. The Early Treatment Diabetic Retinopathy Study grading scale was used to evaluate the retinopathy status. Systemic vascular comorbidities included diabetes mellitus (DM), high blood pressure (HBP), chronic kidney disease (CKD) and cardiovascular disease (CVD). Time to death was calculated as the time from baseline to either the date of death or censoring (December 31^st^, 2015), whichever came first. Risks of mortality were estimated using Cox proportional hazards models after adjusting for confounders and vascular comorbidities.

**Results:**

After a median follow-up of 8.33 years (IQR: 7.50-9.67 years), there were 949 (11.8%) deaths from all causes. After adjusting for confounders, the presence of retinopathy predicted higher all-cause mortality (hazard ratio (HR), 1.41; 95% confidence interval (CI), 1.08-1.83). The all-cause mortality among participants with both retinopathy and systemic vascular comorbidities including DM (HR, 1.72; 95% CI, 1.21-2.43), HBP (HR, 1.47; 95% CI, 1.03-2.10), CKD (HR, 1.73; 95% CI, 1.26-2.39) and CVD (HR, 1.92; 95% CI, 1.21-3.04) was significantly higher than that among those without either condition. When stratified by diabetic or hypertension status, the co-occurrence of retinopathy and CKD or CVD further increased the all-cause mortality compared to those without either condition.

**Conclusions:**

The co-occurrence of retinopathy and systemic vascular conditions predicted a further increase in the risk of mortality. More extensive vascular risk factor assessment and management are needed to detect the burden of vascular pathologies and improve long-term survival in individuals with retinopathy.

## Introduction

Retinopathy commonly refers to a spectrum of signs on the fundus (e.g. microaneurysms, soft/hard exudates, and/or retinal hemorrhage) that are common in the elderly, even in those without diabetes (1). A recent pooled analysis of 22,896 individuals with diabetes reported that nearly 35% had retinopathy (2). Furthermore, previous evidence suggested that up to 15% of non-diabetic patients exhibited clinical signs of retinopathy (3–7).

Previous literature has consistently described significant associations between retinopathy and systemic vascular comorbidities, including diabetes mellitus (DM) (5), high blood pressure (HBP) (8), chronic kidney disease (CKD) (6, 9–11) and cardiovascular disease (CVD) (12–15). The presence of retinopathy has been reported to be an independent risk factor for mortality (16–27). A recent meta-analysis of 20 observational studies reported that individuals with retinopathy had a two- to four-fold increase in their risks of all-cause mortality independent of other potential risk factors (28).

To date, only a few studies have investigated the joint effects of retinopathy and systemic vascular comorbidities [such as DM (16), CKD (16, 29–31), and clinical cerebrovascular disease (16)] on mortality. These previous studies were subject to selection bias, underestimation of retinopathy, short duration of follow-up and a lack of investigation of the effects of concomitant macrovascular disorders. Given that the retina is readily viewable *via* non-invasive retinal imaging and that there is a well-established association between retinopathy and systemic vascular comorbidities, clarifying the precise effects of the co-occurrence of retinopathy and systemic vascular comorbidities on the risk of mortality is of great significance.

The National Health and Nutrition Examination Survey (NHANES) is a continuous, national population-based study of elderly participants in the U.S., which provides a unique opportunity to investigate the impact of retinopathy and systemic vascular comorbidities on mortality.

## Methods

### Sample and Population

The NHANES dataset from 2005 to 2008 was used for the current analysis. NHANES is a nationally representative survey of the non-institutionalized U.S. civilian population that includes in-person interviews and extensive clinical examinations. As described in detail elsewhere, the NHANES uses a multistage design to select participants from strata and proportions of minority populations (32). Since 1999, the NHANES has released a dataset every two years. Because of the availability of retinal imaging, two NHANES cycles (2005-2006 and 2007-2008) were merged. In total, 5,703 out of 6,797 individuals aged 40 years and older were included for the current analysis. A total of 1,093 participants were excluded due to missing information on the grading of retinopathy for both eyes. One participant was excluded because of the lack of mortality data.

The research adhered to the tenets of the Declaration of Helsinki. Written informed consent was given by every participant, and the NHANES was conducted in accordance with ethical standards. Because data used in this analysis were publicly available and de-identified, the present study received exemption from review by the institutional review board.

### Evaluation of Retinopathy

The Canon CR6-45NM Ophthalmic Digital Imaging System and Canon EOS 10D digital camera (Canon USA Inc., One Canon Park, Melville, New York) were used to capture non-mydriatic retinal photographs. Two digital images per eye (one for the macula and one for the optic nerve) were taken in an almost completely dark room. Based on a modification of the Airlie House classification system (33, 34), graders in the University of Wisconsin Ocular Epidemiologic Reading Center, Madison assessed the fundus photographs. Details of image capture and grading of fundus photographs have been described in an earlier study (35). The Early Treatment Diabetic Retinopathy Study (ETDRS) grading scale was used to evaluate the retinopathy severity (33). Participants were classified as having retinopathy (level 14-51) based on the eye with the worse retinopathy level.

### Assessment of Systemic Vascular Comorbidities

Systemic vascular comorbidities, including DM, HBP, CKD and self-reported history of CVD, were assessed. Identification of DM was based on self-reported medical history, use of insulin or antihyperglycaemic medications, or a glycosylated hemoglobin level exceeding 6.5% (36). Identification of HBP was based on a self-reported physician diagnosis, use of antihypertensive medications, or the average of three measurements of systolic ≥140 mmHg) and/or the average of three measurements of diastolic blood pressure ≥90 mmHg). According to the recent American Heart Association classification of HBP, we considered stage 2 HBP in the present analysis (37). An estimated glomerular filtration rate (eGFR) of less than 60 ml/min/1.73 m^2^ was used to as the definition for CKD (38). A self-reported physician diagnosis of coronary heart disease, myocardial infarction, congestive heart failure, angina or stroke was used to identify the self-reported history of CVD.

### Mortality Data

Based on a probabilistic matching algorithm, mortality was ascertained from the National Death Index (NDI) (39). This linkage was performed by matching participants’ personal identification information, such as name, sex, date of birth, social security number, between the NHANES and the NDI datasets. Participants not found to be deceased were considered to be alive. The time between the date of the interview and the date of death or end-of-study censoring (the 31^st^ of December, 2015), whichever came first, was calculated as the duration of follow-up.

### Covariates

Gender (male and female) was included as a covariate. Ethnicity was classified into four groups: non-Hispanic white, non-Hispanic black, Mexican American, and other. Two categories of marital status included unmarried or other, and married or lived with a partner. Education level was classified into two groups: did not complete a high school degree, and completed at least a high school degree. The poverty-income ratio (PIR) was categorized into two groups: < 1.00 and ≥ 1.00 (poverty threshold) respectively. Smoking status was classified into two groups: never, and former or current smokers. Drinking status was classified into two groups: abstainer or former drinker, and current drinker. Identification of hypercholesterolaemia was based on the total cholesterol level (≥240 mg/dL) or the use of antihyperlipidemic agents. Body mass index (BMI) was a value derived from the weight in kilograms divided by the height in meters squared. A high C-reactive protein (CRP) was defined as CRP level ≥1 mg/dL. Walking disability was based on self-reported responses to the questionnaire or the requirement of special equipment to aid walking. Health status was dichotomously classified into poor or fair, and good or excellent.

### Statistical Analysis

The complex, stratified design of NHANES was taken into account for all analyses. The means and standard errors (SEs), or numbers and weighted proportions were used to present the baseline characteristics of the study population where appropriate. The design-adjusted t-test or the Rao-Scott Pearson χ^2^ were used to compare distributions for continuous or categorical variables. We used Kaplan-Meier estimates to generate plots of survival curves among participants with retinopathy and concomitant systemic vascular disorders, and log-rank tests to compare survival distributions among these groups. We used Cox proportional hazards regression models to estimate hazard ratios (HRs) and 95% confidence intervals (CIs) for survival. Furthermore, we explored the joint effects of retinopathy and systemic vascular diseases on mortality. We tested the proportional hazard assumption for each variable in the Cox regression models by creating the time-dependent covariate (interaction between the variable and survival time), with a p-value < 0.05 for the time-dependent covariate indicating a violation of the assumption. We found that all variables included in the Cox regression models were valid. Collinearity among variables was tested using the variance inflation factor (VIF) procedure, and the average value was 1.26 in the present analysis. We used Stata (ver. 14.0; StataCorp., College Station, TX) to perform all analyses. A p-value of less than 0.05 was considered statistically significant.

## Results

In total, there were 6,797 individuals aged 40 years and older from the NHANES 2005-2008. Of these participants, 5,703 were included in the current analysis due to missing information on the grading of retinopathy for both eyes in 1,093 participants and missing data on vital status in 1 participant. Participants with missing data were more likely to be older, black, poorly educated, unmarried or other, have a lower level of household income and be unhealthier in terms of systemic comorbidities when compared to those with complete data. The characteristics of the excluded and the included participants are shown in **Supplement Table 1**.

A total of 710 participants were identified as having retinopathy (weighted prevalence: 9.75%). The mean age of these participants was 56.5 ± 0.38 years (SE), and 52.6% were women. The baseline characteristics of participants by retinopathy status are presented in **Table 1**. Participants identified as having retinopathy were more likely to be older, male, black, poorly educated, and lifetime abstainers/former drinkers and to have higher BMI, proportion of walking disability, poor/fair self-rated health status, and concomitant micro- and macrovascular pathologies (DM, HBP, CKD and CVD) compared to those without retinopathy. No significant difference in other characteristics was observed between these two groups.

**Table 1:** Demographic, health-related behaviors and general health characteristics of participants with and without retinopathy.

Characteristics	Overall, N = 5703	No Retinopathy, n = 4993 (%)	Any Retinopathy, n = 710 (%)	Unadjusted P Value^a^
Age (SE), yrs	56.5 ± 0.38	56.1 ± 0.37	60.1 ± 0.77	**<0.001**
Gender				
Male	2855 (47.4)	2466 (46.6)	389 (55.0)	**<0.001**
Female	2848 (52.6)	2527 (53.4)	321 (45.0)	
Race				
Non-Hispanic white	3059 (77.1)	2761 (78.1)	298 (67.8)	**<0.001**
Non-Hispanic black	1174 (9.66)	963 (9.00)	211 (15.8)	
Mexican American	884 (5.43)	757 (5.24)	127 (7.19)	
Other	586 (7.83)	512 (7.68)	74 (9.24)	
Education				
Less than high school	1682 (18.1)	1405 (17.3)	277 (25.8)	**<0.001**
High school and over	4021 (81.9)	3588 (82.7)	433 (74.2)	
Marital status				
Unmarried and other	2071 (31.1)	1804 (30.9)	267 (32.4)	0.430
Married/with a partner	3629 (68.9)	3186 (69.1)	443 (67.6)	
Poverty income ratio (PIR)				
Below poverty (<1)	841 (9.29)	729 (9.21)	112 (10.0)	0.489
At or above poverty (≥1)	4460 (90.7)	3914 (90.8)	546 (90.0)	
Smoking status				
Never	2687 (48.3)	2347 (48.4)	340 (47.6)	0.770
Former/Current	3012 (51.7)	2642 (51.6)	370 (52.4)	
Alcohol consumption				
Lifetime abstainer/former drinker	1380 (20.6)	1158 (19.4)	222 (31.1)	**<0.001**
Current drinker	4181 (79.4)	3710 (80.6)	471 (68.9)	
Hypercholesterolaemia				
No	2606 (48.4)	2326 (48.9)	280 (43.8)	0.096
Yes	2940 (51.6)	2530 (51.1)	410 (56.2)	
BMI (SE), kg/m^2^	29.1 ± 0.14	29.0 ± 0.15	30.0 ± 0.27	**0.001**
High C-reactive protein				
No	4876 (89.4)	4282 (89.4)	594 (89.1)	0.840
Yes	634 (10.6)	547 (10.6)	87 (10.9)	
Walking disability				
No	5088 (91.7)	4491 (92.4)	597 (86.0)	**<0.001**
Yes	615 (8.26)	502 (7.64)	113 (14.0)	
Self-rated health				
Poor/Fair	1466 (18.8)	1191 (17.4)	275 (32.2)	**<0.001**
Good/Excellent	4111 (81.2)	3692 (82.6)	419 (67.8)	
DM				
No	4432 (85.7)	4091 (88.6)	341 (58.2)	**<0.001**
Yes	1129 (14.4)	772 (11.4)	357 (41.8)	
HBP				
No	2784 (56.2)	2565 (57.8)	219 (41.2)	**<0.001**
Yes	2824 (43.8)	2356 (42.2)	468 (58.8)	
CKD				
No	4088 (80.5)	3704 (82.2)	384 (65.0)	**<0.001**
Yes	1413 (19.5)	1113 (17.8)	300 (35.0)	
CVD				
No	4796 (87.8)	4285 (89.0)	511 (76.2)	**<0.001**
Yes	907 (12.2)	708 (11.0)	199 (23.8)	

SE, standard error; BMI, body mass index; DM, diabetes mellitus; HBP, high blood pressure; CKD, chronic kidney disease; CVD, cardiovascular disease. Boldface indicates statistical significance.

All proportions are weighted estimates of the US population characteristics, taking into account the complex sampling design of the National Health and Nutrition Examination Survey.

a All P values were calculated using t-test for continuous variables and the design-adjusted Rao-Scott Pearson χ^2^ test for categorical variables.

The median duration of follow-up was 8.33 years (interquartile range [IQR]: 7.50-9.67 years). There were 949 (11.8%) deaths from all causes. Participants with retinopathy had higher all-cause mortality than those without (23.0% *versus* 10.5%, t-test P < 0.001). The baseline characteristics, including demographic factors, health-related behaviors and general health characteristics for participants by survival status, are presented in **Table 2**. Several covariates, including age, sex, ethnicity, educational attainment, types of marital status, household income level, smoking status, alcohol consumption, the level of CRP, self-rated health status, walking disability, and micro- and macrovascular disorders (DM, CKD and history of CVD), were strongly associated with all-cause mortality in age- and sex-adjusted models. Multivariate Cox regression models suggested that the presence of any degree of retinopathy at baseline was associated with higher all-cause mortality (HR, 1.41; 95% CI, 1.08-1.83; P=0.012).

**Table 2:** All-cause mortality by demographic, health-related behaviors and general health characteristics.

Characteristics	No. of Living Subjects, n = 4754 (%)	No. of Deceased Subjects, n = 949 (%)	HR (95% CI)^a^
Age (SE), yrs	54.9 ± 0.35	68.7 ± 0.57	**1.10 (1.09-1.11)**
Gender			
Male	2294 (46.7)	561 (52.7)	Reference
Female	2460 (53.3)	388 (47.3)	**0.68 (0.58-0.81)**
Race			
Non-Hispanic white	2470 (76.8)	589 (78.8)	Reference
Non-Hispanic black	977 (9.52)	187 (10.7)	**1.55 (1.31-1.83)**
Mexican American	779 (5.55)	105 (4.54)	**1.30 (1.02-1.67)**
Other	528 (8.09)	58 (5.92)	1.12 (0.71-1.76)
Education			
Less than high school	1290 (16.1)	392 (33.6)	Reference
High school and over	3464 (83.9)	557 (66.4)	**0.57 (0.48-0.68)**
Marital status			
Unmarried and other	1627 (29.2)	444 (45.3)	Reference
Married/with a partner	3124 (70.8)	505 (54.7)	**0.62 (0.54-0.72)**
Poverty income ratio (PIR)			
Below poverty (<1)	650 (8.39)	191 (16.3)	Reference
At or above poverty (≥1)	3781 (91.6)	679 (83.7)	**0.44 (0.34-0.56)**
Smoking status			
Never	2334 (50.1)	353 (35.1)	Reference
Former/Current	2417 (49.9)	595 (64.9)	**1.64 (1.38-1.95)**
Alcohol consumption			
Lifetime abstainer/former drinker	1094 (19.3)	286 (30.6)	Reference
Current drinker	3549 (80.7)	632 (69.4)	**0.73 (0.63-0.84)**
Hypercholesterolaemia			
No	2195 (48.7)	411 (45.7)	Reference
Yes	2445 (51.3)	495 (54.3)	0.88 (0.75-1.02)
BMI (SE), kg/m^2^	29.2 ± 0.15	28.2 ± 0.22	0.99 (0.97-1.01)
High C-reactive protein			
No	4137 (90.2)	739 (82.9)	Reference
Yes	481 (9.76)	153 (17.1)	**2.05 (1.49-2.81)**
Walking disability			
No	4386 (94.1)	702 (74.1)	Reference
Yes	368 (5.91)	247 (25.9)	**2.77 (2.26-3.40)**
Self-rated health			
Poor/Fair	1086 (16.2)	380 (38.4)	Reference
Good/Excellent	3570 (83.8)	541 (61.6)	**0.38 (0.33-0.44)**
DM			
No	3796 (87.3)	636 (73.0)	Reference
Yes	848 (12.7)	281 (27.0)	**1.75 (1.40-2.19)**
HBP			
No	2490 (59.1)	294 (34.0)	Reference
Yes	2201 (40.9)	623 (66.0)	**1.31 (1.07-1.61)**
CKD			
No	3658 (84.5)	430 (50.5)	Reference
Yes	938 (15.5)	475 (49.5)	**1.93 (1.64-2.26)**
CVD			
No	4195 (90.9)	601 (63.9)	Reference
Yes	559 (9.07)	348 (36.1)	**2.17 (1.88-2.49)**

SE, standard error; BMI, body mass index; DM, diabetes mellitus; HBP, high blood pressure; CKD, chronic kidney disease; CVD, cardiovascular disease. Boldface indicates statistical significance.

All-cause mortality was assessed through December 31, 2015. All proportions, means and standard errors are weighted estimates of the US population characteristics, taking into account the complex sampling design of the National Health and Nutrition Examination Survey.

a Adjusted for age and gender.

**Figure 1:**
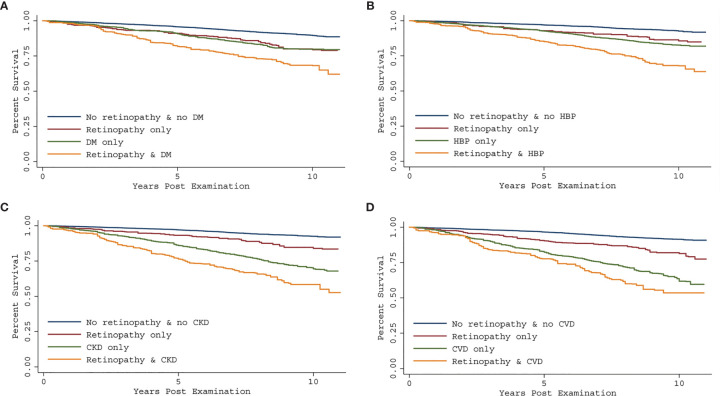
Kaplan-Meier curve showing all-cause mortality rate by retinopathy status and concomitant systemic vascular disorders [**(A)**: diabetes mellitus; **(B)**: high blood pressure; **(C)**: chronic kidney disease] **(D)**: history of cardiovascular disease), using the 2005-2008 National Health and Nutrition Examination Survey (NHANES). All-cause mortality was assessed through December 31, 2015. Risk of mortality was further increased among those with both retinopathy and systemic vascular diseases compared to participants without either condition.

Interactions between retinopathy and each systemic vascular disorder were assessed, and no significant interaction for DM, CKD, or CVD was found. There was a significant interaction between retinopathy and HBP in the model (P=0.041). Kaplan-Meier curves for all-cause mortality according to retinopathy and systemic vascular conditions are shown in **Figure 1**. **Table 3** presents the synergistic impact of each vascular disorder together with retinopathy on mortality. All-cause mortality for participants with retinopathy and comorbidities such as DM (HR, 1.72; 95% CI, 1.21-2.43; P=0.003), HBP (HR, 1.47; 95% CI, 1.03-2.10; P=0.034), CKD (HR, 1.73; 95% CI, 1.26-2.39; P=0.002) and CVD (HR, 1.92; 95% CI, 1.21-3.04; P=0.007) was significantly higher than for those without any condition.

**Table 3:** Cox proportional hazards regression models of all-cause and cardiovascular disease mortality by retinopathy status and concomitant medical conditions.

	No. of Events	Mortality Rate^a^	Model 1^b^	Model 2^c^	P for Interaction^d^
Retinopathy and DM status					0.572
Neither retinopathy nor DM (N = 4091)	563	9.36%	1.00 (reference)	1.00 (reference)	
Retinopathy only (N = 341)	73	17.9%	**1.70 (1.33-2.16)**	**1.49 (1.16-1.90)**	
DM only (N = 772)	167	18.6%	**1.52 (1.15-2.00)**	**1.30 (1.03-1.66)**	
Both DM and retinopathy (N = 357)	114	30.3%	**2.17 (1.54-3.06)**	**1.72 (1.21-2.43)**	
Retinopathy and HBP status					**0.041**
Neither retinopathy nor HBP (N = 2565)	253	6.56%	1.00 (reference)	1.00 (reference)	
Retinopathy only (N = 219)	41	12.5%	**2.04 (1.35-3.10)**	**2.08 (1.30-3.31)**	
HBP only (N = 2356)	485	15.6%	**1.28 (1.03-1.60)**	1.19 (0.93-1.52)	
Both HBP and retinopathy (N = 468)	138	29.1%	**1.98 (1.44-2.70)**	**1.47 (1.03-2.10)**	
Retinopathy and CKD status					0.063
Neither retinopathy nor CKD (N = 3704)	370	6.71%	1.00 (reference)	1.00 (reference)	
Retinopathy only (N = 384)	60	13.9%	**1.83 (1.27-2.64)**	**1.71 (1.20-2.43)**	
CKD only (N = 1113)	353	27.3%	**1.82 (1.55-2.14)**	**1.42 (1.14-1.77)**	
Both CKD and retinopathy (N = 300)	122	39.5%	**2.74 (2.04-3.69)**	**1.73 (1.26-2.39)**	
Retinopathy and CVD status					0.062
Neither retinopathy nor CVD (N = 4285)	502	7.80%	1.00 (reference)	1.00 (reference)	
Retinopathy only (N = 511)	99	16.7%	**1.82 (1.39-2.38)**	**1.71 (1.30-2.24)**	
CVD only (N = 708)	257	32.7%	**2.07 (1.79-2.39)**	**1.75 (1.45-2.11)**	
Both CVD and retinopathy (N = 199)	91	43.1%	**2.85 (2.02-4.03)**	**1.92 (1.21-3.04)**	

DM, diabetes mellitus; HBP, high blood pressure; CKD, chronic kidney disease; CVD, cardiovascular disease.

Boldface indicates statistical significance. Values are hazard ratios (95% confidence interval).

All-cause mortality was assessed through December 31, 2015.

a All proportions are weighted estimates of the US population characteristics, taking into account the complex sampling design of the National Health and Nutrition Examination Survey.

b Model 1: Adjusted for age, gender, race, education level, marital status, income status.

c Model 2: Model 1 plus additional adjustments for BMI, smoking status, drinking status, hypertension, diabetes mellitus, hypercholesterolaemia, C-reactive protein, self-rated health status, walking disability, self-reported history of cardiovascular disease and chronic kidney disease.

d Interaction between retinopathy and either DM, HBP, CKD, or CVD.

The results stratified by diabetes and hypertension status are shown in **Supplement Table 2**. Among non-diabetic participants, all-cause mortality was higher in those with retinopathy and CKD (HR, 1.55; 95% CI, 1.07-2.25; P=0.023). Among diabetic participants, the co-occurrence of retinopathy and CVD (HR, 2.52; 95% CI, 1.20-5.29; P=0.016) further increased all-cause mortality. For participants without hypertension, the co-occurrence of retinopathy and CVD posed a higher risk of death (HR, 3.32; 95% CI, 1.32-8.35; P=0.012). In an analysis limited to participants with hypertension, the joint effect of retinopathy and CVD (HR, 1.64; 95% CI, 1.01-2.67; P=0.047) or CKD (HR, 1.85; 95% CI, 1.35-2.55; P<0.001) predicted higher all-cause mortality than that of those without either condition.

## Discussion

In this large sample of middle-aged and older adults, participants with retinopathy had a higher all-cause mortality rate. Moreover, the co-occurrence of retinopathy and systemic vascular conditions (DM, HBP, CKD, and history of CVD) further increased all-cause mortality.

Our findings are in line with previous results that reported a significant association between retinopathy and mortality (17–26). Contrary to the EURODIAB Prospective Complications Study, which found that the relationship between retinopathy and mortality can be largely explained by the presence of coexisting cardiovascular risk factors (40), we report that retinopathy was an independent risk factor for mortality after comprehensive adjustment of covariates. We postulate that the observed difference might be a result of missing fundus photographs (31%) and morbidity data (14%) in the EURODIAB Prospective Complications Study, which may have led to the underestimation of the association between retinopathy and mortality.

Only a limited number of studies have attempted to explore the joint effects of retinopathy and systemic vascular comorbidities [such as DM (16), CKD (16, 29–31), and clinical cerebrovascular disease (16)] on mortality. A more detailed investigation of systemic vascular comorbidities (DM, HBP, CKD, and CVD) in our study provides further insights into the association between retinopathy and mortality. That is, our results not only support previous findings of a higher mortality risk in individuals with retinopathy but also suggest that retinopathy in the presence of concomitant DM, HBP, CKD or CVD increases the risk of mortality even further.

Mechanisms underlying the association between retinopathy and mortality remain unknown. It has been proposed that retinopathy may be an indicator of the burden of CVD risk factors, including dyslipidemia, obesity, hypertension, and smoking (17). However, our results do not support this theory. Although we found that the distribution of CVD risk factors differed between non-retinopathy and retinopathy groups, retinopathy was found to be an independent risk factor for mortality in the fully adjusted model. Another potential explanation is that retinopathy, as a marker of abnormal microcirculation in the retina, may be an important indicator of systemic micro- and macro-vascular abnormalities (24). These micro- and macrovascular disorders may share similar pathophysiological processes with retinopathy, including endothelial dysfunction, inflammation, apoptosis, and neovascularization (41, 42). In addition, the co-occurrence of retinopathy and systemic vascular disorders may reflect a greater burden and/or severity of vascular pathologies, which may explain the further increase in mortality among the participants who have both retinopathy and systemic vascular disorders, and the marginally interaction between retinopathy and HBP.

Given that retinopathy can be readily detected *via* non-invasive means, our findings may have several practical implications. Firstly, our finding of a statistically significant association between retinopathy and systemic vascular abnormalities indicates that individuals with retinopathy may benefit from a comprehensive vascular assessment and should be closely monitored. Secondly, the current study and previous studies find that the co-occurrence of retinopathy and micro- or macrovascular disorders poses a further increase in all-cause mortality. Therefore, intensive vascular risk reduction may be warranted in the management of these patients.

The strengths of this analysis include the use of a large-scale sample with national representativeness, the use of a standardized objective grading protocol to assess retinopathy, access to death records, long-term follow-up and inclusion of a comprehensive range of covariates. However, several potential limitations should be considered. Firstly, retinopathy status and potential confounders adjusted for in our analysis were assessed on a single occasion. During the follow-up period, the behaviors and/or retinopathy status of the patients may have changed, which may have a direct impact on the outcome. Secondly, discrepancies may exist between the self-reported data and clinically measured data. This may cause underreporting of milder cases and subsequently lead to a bias in the analysis. Finally, participants with missing data tended to be in poorer health status, which may have resulted in selection bias.

In summary, our findings suggest that middle-aged and elderly people with retinopathy have increased all-cause mortality. Furthermore, the joint effects of retinopathy and major systemic vascular comorbidities increase the all-cause mortality further. Our results indicate that more extensive risk factor assessment and management of individuals with retinopathy may be beneficial to reduce their mortality rate, especially in patients who have both retinopathy and systemic vascular disorders.

## Data Availability Statement

Publicly available datasets were analyzed in this study. This data can be found here: https://www.cdc.gov/nchs/nhanes/index.htm.

## Author Contributions

Study concept and design: ZZ, MH, and XY. Acquisition, analysis, or interpretation: All authors. Drafting of the manuscript: ZZ and XS. Critical revision of the manuscript for important intellectual content: WW, JH, YC, MH, and XY. Statistical analysis: ZZ, XS, and WW. Obtained funding: MH. Administrative, technical, or material support: ZZ, XS, WW, MH, and XY. Study supervision: MH and XY. All authors contributed to the article and approved the submitted version.

## Funding

The present work was supported by Fundamental Research Funds of the State Key Laboratory of Ophthalmology (303060202400362), National Natural Science Foundation of China (82000901, 82101173), Project of Special Research on Cardiovascular Diseases (2020XXG007), and Research Foundation of Medical Science and Technology of Guangdong Province (B2021237). MH receives support from the University of Melbourne at Research Accelerator Program and the CERA Foundation. The Centre for Eye Research Australia receives Operational Infrastructure Support from the Victorian State Government. The sponsor or funding organization had no role in the design or conduct of this research.

## Conflict of Interest

The authors declare that the research was conducted in the absence of any commercial or financial relationships that could be construed as a potential conflict of interest.

## Publisher’s Note

All claims expressed in this article are solely those of the authors and do not necessarily represent those of their affiliated organizations, or those of the publisher, the editors and the reviewers. Any product that may be evaluated in this article, or claim that may be made by its manufacturer, is not guaranteed or endorsed by the publisher.

## Abbreviations

CVD, cardiovascular disease; NHANES, National Health and Nutrition Examination Survey; DM, diabetes millitus; HBP, high blood pressure; CKD, chronic kidney disease; HR, hazard ratios; CI, confidence interval; ETDRS, Early Treatment Diabetic Retinopathy Study; NDI, National Death Index; eGFR, estimated glomerular filtration rate; ICD, International Classification of Diseases, Injuries and Causes of death; BMI, body mass index; CRP, C-reactive protein; PIR, poverty income ratio; SE, standard errors; VIF, variance inflation factors; IQR, interquartile range.
