# Supplementary material for: Impact of Retinopathy and Systemic Vascular Comorbidities on All-Cause Mortality
Source: Front Endocrinol (Lausanne). 2021 Nov 18;12:750017. doi: 10.3389/fendo.2021.750017 (PMC8637619; doi:10.3389/fendo.2021.750017)
Supplement: Supplementary file 1 [file DataSheet_1.pdf]

Supplement Table 1. Demographic, Health-Related Behaviors and General Health Characteristics of Participants Included and Excluded in the Analysis

| Characteristics | No. of Excluded Subjects, n = 1094 (%) | No. of Included Subjects, n = 5703 (%) | P Value [a] |
|---|---|---|---|
| Age (SE), yrs | 60.9 (0.72) | 56.5 (0.38) | **<0.001** |
| Gender | | | |
| Male | 518 (45.0) | 2855 (47.4) | 0.226 |
| Female | 576 (55.0) | 2848 (52.6) | |
| Race | | | |
| Non-Hispanic white | 487 (65.7) | 3059 (77.1) | **<0.001** |
| Non-Hispanic black | 295 (16.1) | 1174 (9.66) | |
| Mexican American | 164 (6.16) | 884 (5.43) | |
| Other | 148 (12.0) | 586 (7.83) | |
| Education | | | |
| Less than high school | 459 (28.9) | 1682 (18.1) | **<0.001** |
| High school and over | 635 (71.1) | 4021 (81.9) | |
| Marital status | | | |
| Unmarried and other | 505 (38.8) | 2071 (31.1) | **0.001** |
| Married/with a partner | 586 (61.2) | 3629 (68.9) | |
| Poverty income ratio (PIR) | | | |
| Below poverty (<1) | 203 (13.9) | 841 (9.29) | **0.008** |
| At or above poverty (≥1) | 764 (86.1) | 4460 (90.7) | |
| Smoking status | | | |
| Never | 608 (55.6) | 2687 (48.3) | **0.002** |
| Former/Current | 483 (44.4) | 3012 (51.7) | |
| Alcohol consumption | | | |
| Lifetime abstainer/former drinker | 255 (34.1) | 1380 (20.6) | **<0.001** |

| | | | |
|---|---|---|---|
| Current drinker | 426 (65.9) | 4181 (79.4) | |
| High cholesterol | | | |
| No | 456 (47.5) | 2606 (48.4) | 0.632 |
| Yes | 502 (52.5) | 2940 (51.6) | |
| BMI (SE), kg/m$^2$ | 28.5 (0.27) | 29.1 (0.14) | 0.117 |
| High C-reactive protein | | | |
| No | 796 (88.2) | 4876 (89.4) | 0.418 |
| Yes | 123 (11.8) | 634 (10.6) | |
| Walking disability | | | |
| No | 807 (78.0) | 5088 (91.7) | **<0.001** |
| Yes | 287 (22.0) | 615 (8.26) | |
| Self-rated health | | | |
| Poor/Fair | 264 (31.2) | 1466 (18.8) | **<0.001** |
| Good/Excellent | 428 (68.8) | 4111 (81.2) | |
| DM | | | |
| No | 669 (75.1) | 4432 (85.7) | **<0.001** |
| Yes | 295 (24.9) | 1129 (14.4) | |
| HBP | | | |
| No | 349 (46.1) | 2784 (56.2) | **<0.001** |
| Yes | 515 (53.9) | 2824 (43.8) | |
| CKD | | | |
| No | 507 (64.3) | 4088 (80.5) | **<0.001** |
| Yes | 385 (35.7) | 1413 (19.5) | |
| CVD | | | |
| No | 833 (79.6) | 4796 (87.8) | **<0.001** |
| Yes | 261 (20.4) | 907 (12.2) | |

Abbreviations: SE, standard error; BMI, body mass index; DM, diabetes mellitus; HBP, high blood pressure; CKD, chronic kidney disease; CVD, cardiovascular disease.

All proportions are weighted estimates of the US population characteristics, taking into account the complex sampling design of the National Health and Nutrition Examination Survey.

[a] All P values were calculated using the t-test for continuous variables and design-adjusted Rao-Scott Pearson $\chi^2$ test for categorical variables. Boldface indicates statistical significance.

Supplement Table 2. Stratified Analysis of Cox Proportional Hazards Regression Models of All-Cause and Cardiovascular Disease Mortality by Retinopathy Status and Concomitant Medical Conditions.

| | **All-Cause Mortality** | | | |
|---|---|---|---|---|
| | **DM** | | **HBP** | |
| | **No (N = 4432)** | **Yes (N = 1129)** | **No (N = 2784)** | **Yes (N = 2824)** |
| Retinopathy and CKD status | | | | |
| Neither retinopathy nor CKD | 1.00 (reference) | 1.00 (reference) | 1.00 (reference) | 1.00 (reference) |
| Retinopathy only | **1.99 (1.32-2.99)** | 1.21 (0.52-2.84) | **1.82 (1.17-2.81)** | **1.65 (1.04-2.61)** |
| CKD only | **1.41 (1.12-1.76)** | 1.35 (0.80-2.27) | 0.82 (0.60-1.12) | **1.78 (1.32-2.39)** |
| Both CKD and retinopathy | **1.55 (1.07-2.25)** | 1.74 (0.94-3.21) | 2.05 (0.73-5.80) | **1.85 (1.35-2.55)** |
| Retinopathy and CVD status | | | | |
| Neither retinopathy nor CVD | 1.00 (reference) | 1.00 (reference) | 1.00 (reference) | 1.00 (reference) |
| Retinopathy only | **1.83 (1.31-2.56)** | 1.66 (0.80-3.44) | **2.03 (1.17-3.53)** | 1.41 (0.99-2.01) |
| CVD only | **1.51 (1.20-1.89)** | **2.50 (1.51-4.15)** | **1.93 (1.19-3.14)** | **1.58 (1.15-2.19)** |
| Both CVD and retinopathy | 1.56 (0.88-2.78) | **2.52 (1.20-5.29)** | **3.32 (1.32-8.35)** | **1.64 (1.01-2.67)** |

Abbreviations: DM, diabetes millitus; HBP, high blood pressure; CKD, chronic kidney disease; CVD, cardiovascular disease.

Boldface indicates statistical significance. Values are number of hazard ratio (95% confidence interval).

All-cause mortality was assessed through December 31, 2015.

All proportions are weighted estimates of the US population characteristics, taking into account the complex sampling design of the National Health and Nutrition Examination Survey.

[a] Adjusted for age, gender, race, education level, marital status, income status, BMI, smoking status, drinking status, hypertension, diabetes millitus, cholesterol level, C-reactive protein, self-rated health status, walking disability, self-reported history of cardiovascular disease and chronic kidney disease.
